# An Octopus-Inspired Soft Pneumatic Robotic Arm

**DOI:** 10.3390/biomimetics9120773

**Published:** 2024-12-19

**Authors:** Emmanouil Papadakis, Dimitris P. Tsakiris, Michael Sfakiotakis

**Affiliations:** 1 Institute of Computer Science, Foundation for Research and Technology–Hellas, GR-70013 Heraklion, Greece; tsakiris@ics.forth.gr; 2 Department of Electrical and Computer Engineering, Hellenic Mediterranean University, GR-71410 Heraklion, Greece; msfak@hmu.gr

**Keywords:** soft robotics, biomimetics, bio-inspired robots, robotic arm, pneumatic actuation, robot control, arm twisting

## Abstract

This paper addresses the design, development, control, and experimental evaluation of a soft robot arm whose actuation is inspired by the muscular structure of the octopus arm, one of the most agile biological manipulators. The robot arm is made of soft silicone and thus possesses enhanced compliance, which is beneficial in a variety of applications where the arm may come into contact with delicate features of its environment. The arm is composed of three elongated segments arranged in series, each one of which contains several pneumatically actuated chambers embedded in its silicone body, which may induce various types of deformations of the segment. By combining the segment deformations, and by imitating the antagonistic muscle group functionality of the octopus, the robot arm can bend in various directions, increase or decrease its length, as well as twist around its central axis. This is one of the few octopus-inspired soft robotic arms where twisting is replicated in its motion characteristics, thus greatly expanding the arm’s potential applications. We present the design process and the development steps of the soft arm, where the molding of two-part silicone of low hardness in 3d-printed molds is employed. In addition, we present the control methodology and the experimental evaluation of both a standalone segment and the entire three-segment arm. This experimental evaluation involves model-free closed-loop control schemes, exploiting visual feedback from a pair of external cameras in order to reconstruct in real time the shape of the soft arm and the pose of its tip.

## 1. Introduction

The development of robotic systems that offer high flexibility and adaptability, especially in delicate interaction environments, represents a key challenge in robotics research. Conventional robotic systems, characterized by their rigid structures, often encounter difficulties in complex tasks that demand a blend of strength, precision, and adaptability. This challenge is underscored in domains such as underwater exploration, agricultural automation, and medical surgery, where the ability to navigate and manipulate within constrained and/or delicate environments is paramount.

Contemporary endeavors in robotics have progressively embraced bio-inspired designs to overcome the limitations inherent to rigidity, drawing inspiration from the natural world. The muscular hydrostat system of the octopus arm, capable of complex locomotion and manipulation tasks, stands out as a particularly compelling blueprint for soft robotic applications. Early pioneering work in this domain, such as the research by Walker et al. [1], investigated continuum robot arms inspired by cephalopods. This exploration was further expanded upon by studies like [2,3], which demonstrated the potential of leveraging biological principles to enhance robotic flexibility and dexterity by mimicking the cephalopod’s morphology. In [4,5,6], soft robotic grippers and a robotic octopus were developed, exemplifying the feasibility and efficiency of biomimetic approaches in promoting robotic adaptability.

Advancements in material science and novel actuation mechanisms have played a pivotal role in the evolution of soft robotics. The integration of soft sensors within robotic systems [7], alongside foundational work on the design and fabrication of soft robots [8], has paved the way for the development of robots capable of safely interacting with humans and delicate objects. Moreover, the creation of soft robotic fish capable of autonomous underwater navigation [9] underscores the application-specific advantages of soft robotics in challenging environments.

The field has continued to evolve with recent studies pioneering the use of innovative materials and structures to more closely mimic biological functions [10,11,12,13,14,15]. This has opened new avenues for creating more versatile and safer robotic interactions across a broad spectrum of applications, from healthcare to environmental monitoring. Particularly noteworthy is the series of soft robot arms for minimally invasive surgery developed by Menciassi and colleagues (see [16] and references therein). Their work presents the design, fabrication, characterization, and testing of a three-module soft manipulator inspired by the octopus arms and actuated by embedded fluidic actuators, which allow omnidirectional bending and elongation. Furthermore, the stiffness of the manipulator is controlled via a granular-jamming-based stiffening mechanism integrated along the length of the arm. Other arm designs of the same group exploit tendons for tuning the arm stiffness [17]. The development of soft actuators, as explored in [18,19,20,21], further illustrates the potential of soft robotics to transform traditional approaches to robotic design and application.

In addition to material innovations, control strategies for soft robotic systems have seen rapid advancements. Research focusing on the integration of sensory feedback and biomimetic design principles [22,23,24,25,26] has contributed significantly to our understanding of how soft robotic systems can be designed, controlled, and applied in real-world scenarios with a level of sophistication and efficiency akin to their biological counterparts. Together, these studies form a robust foundation for the future of soft robotics, pointing the way toward systems that are not only adaptable and safe but also capable of complex autonomous decision-making and sophisticated environmental interactions. Performance is a critical metric in the field of soft robotics, encompassing attributes such as dexterity, force output, motion range, adaptability, and responsiveness to varying tasks and environments. Metrics such as energy efficiency, actuation speed, load capacity, and control accuracy are commonly used to evaluate the effectiveness of soft robots. By benchmarking these performance indicators [27], soft robotic systems can be optimized for specific use cases, paving the way for advances in functionality and real-world applicability.

Adding twisting ability to a soft robotic arm significantly enhances its versatility and functionality, making it more capable of mimicking the complex motion capabilities of biological systems. While bending and elongation allow for basic directional control and reach, twisting enables the arm to perform additional tasks that require rotational movement. This is particularly important for grasping and manipulating objects with precision, as twisting can adjust the orientation of the end-effector or wrapped arm to better interact with an object. Moreover, twisting greatly expands the arm’s range of potential applications. For example, in minimally invasive surgery, twisting motions are essential for navigating confined spaces and reorienting instruments without requiring external rotation mechanisms. Similarly, in underwater robotics or search-and-rescue scenarios, the ability to twist can allow for intricate interactions with irregularly shaped objects or environments. Twisting also facilitates more natural and efficient movement patterns, reducing the need for complex kinematic configurations or additional joints, therefore simplifying the system while enhancing its adaptability.

Despite these potential advantages, only a limited number of existing soft robotic arm prototypes provide for twisting motions. One such prototype, proposed in [28], utilizes a total of 32 pneumatically driven McKibben actuators to emulate the octopus arm musculature. Four of these actuators are helically wound (two in the clockwise and two in the counterclockwise direction) and can provide torsional motions of the arm through appropriate pressurization. However, the helical actuators form the outer layer of the arm, making them susceptible to breaking if sufficient contact forces are exerted, e.g., from an environment containing sharp edges. In [29], the previous arm is used in conjunction with flexible strain sensors providing information on its bending to implement master-slave feedback control of the arm. In [30], a single-segment pneumatic soft robotic wrist is presented, based on four parallel soft helical actuators, able to generate both bending and twisting movements. In [31], an innovative modular magnetically actuated robot arm based on reconfigurable bistable Kresling origami modules is presented, which can stretch, fold, bend, and twist in various directions. This arm has the advantages of scalability and reconfigurability, but its need for precise magnetic actuation can be seen as a limitation.

In this context, our approach in this paper builds upon these foundational studies and extends our prior work on octopus-inspired robotic systems [3,4,5], aiming to bridge the gap between bio-inspired design principles and the practical requirements of robotic applications. By focusing on the octopus arm’s muscular structure, we have developed a modular soft pneumatic robotic arm that not only demonstrates enhanced compliance and maneuverability but also introduces the ability for twisting movements. The robot arm features linear air chambers mimicking the longitudinal muscles of the octopus arm, enabling bending and elongation, while helical air chambers, replicating the oblique muscles of the octopus, enable twisting motions—a novel feature, not frequently occurring in similar soft robot arms. Compressed air is delivered through silicone tubes routed through the center of the arm, leading to a robust design capable of complex, multi-segment motions. Furthermore, rigid 3d-printed rings were employed to constrain the radial expansion of the arm, similarly to the transverse muscles of the octopus, thus ensuring precise, directed movement, as bending and twisting movements are decomposed and can be controlled independently of each other. The arm also features a conical, overlapping design of its three segments in order to avoid bending dead zones, with appropriately shaped chambers for uniform actuation. The arm prototype was fabricated by casting silicone in 3d printed molds, an involved multi-step process, often prone to inconsistencies. The arm is controlled via simple, model-free closed-loop control schemes, whose efficiency was demonstrated by tip positioning experiments. This experimental evaluation demonstrated the functionality and advantages of the developed soft robot arm.

## 2. Octopus Arm Actuation and Control

The main characteristics of the octopus arm muscular and motion control systems are presented here, to the extent that they are relevant to the design and functionality of our soft robot arm. For a more detailed overview of the octopus arm biomechanics, see, e.g., [32]. The octopus is an ideal animal model for studying elongated flexible appendages, including the generation and control of their movement, as well as the generation of related complex behaviors. The muscular system of the octopus arm consists of a densely packed three-dimensional array of muscle fibers and connective tissue, which extends for the full length of the arm and surrounds a central axial nerve cord. This generates both the forces required for arm movement and deformation, as well as the stiffness variations needed for skeletal-like support. Such a muscular system is called *muscular hydrostat*, and its main characteristic is that it maintains the arm volume constant. Thus, any change in one dimension of the arm will be compensated for by a change in at least one other dimension [33,34].

**Figure 1:**
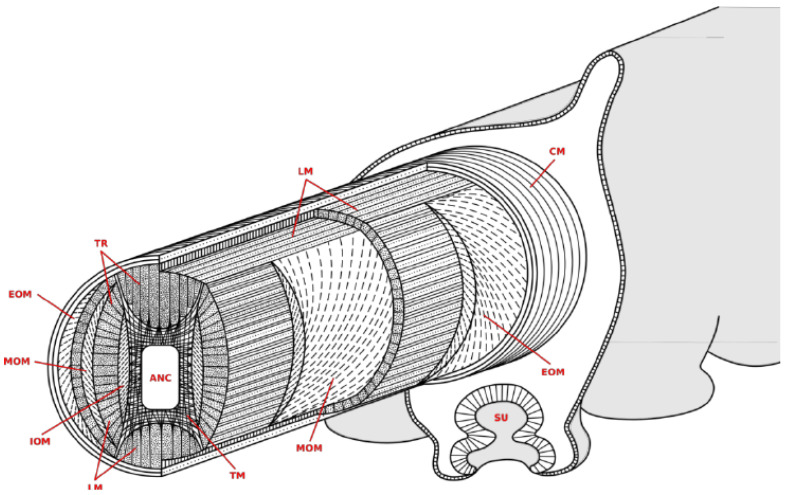
Octopus vulgaris arm muscle system. Image source: [35].

The muscular tissue of each arm is organized into three major groups (Figure 1): The first consists of the longitudinal muscles (LM), oriented parallel to the long axis of the arm and placed in a cross pattern surrounding the central core of the arm. The second group consists of the transverse muscles (TM), which are oriented perpendicular to the long axis of the arm, directly surround the central axial nerve cord, and are surrounded by the longitudinal muscles. The third group consists of the helical or oblique muscles, namely three pairs of helicoidally arranged muscles, the external (EOM), medial (MOM), and internal (IOM) muscles, arranged as a clockwise and a counterclockwise helix along the arm.

A wide variety of, possibly highly localized, arm movements are feasible. The longitudinal muscles are responsible for shortening the arm, the transverse muscles are responsible for its elongation, and the oblique muscles are responsible for torsion. When the transverse muscles are activated, the diameter of the arm is shortened, and since its volume is constant, the arm is elongated. The longitudinal muscles act antagonistically to the transverse ones by shortening the arm and re-extending the transverse muscle fibers. Simultaneous contraction of the longitudinal and the transverse muscles results in stiffening of the arm. Furthermore, select longitudinal and transverse muscles may be simultaneously contracted to bend the arm in all possible directions: as the longitudinal muscles are selectively contracted, the transverse muscles also need to contract in order to resist the arm shortening that would result from this [33,36]. Finally, the oblique muscles are employed in order to twist the arm along its axis, which is accomplished by the controlled activation of these muscle groups, while the direction of torsion, clockwise or counterclockwise, depends on the handedness of the actuated oblique muscle group [35]. These arm deformations can be combined to create more complex movements.

The virtually infinite degrees-of-freedom (dof), which characterize the flexible octopus arms, make efficient control and arm behavior generation quite challenging [37,38,39]. From the engineering point of view, the special strategies and shortcuts employed by these robust and adaptable biological mechanisms may provide valuable hints for addressing corresponding problems in robotic systems [1]. Such strategies include the exploitation of basic “hardwired” motion patterns, like those unveiled in detailed studies of the reaching and fetching primitive movements of the octopus arm. Reaching for a target is implemented by forming a bend near the base of the arm and propagating it towards its tip via a stiffening wave of muscle contraction [39,40,41]. Fetching is implemented by forming a 3-joint-like structure with the arm and rotating the joint-like bends so that the tip reaches the mouth [42]. Control of both of these movements exploits a drastic reduction of the infinite-dof problem via the exploitation of a few basic motion patterns to the control of just three variables [38,39,42,43]. Detailed modeling of the octopus muscular system and computational investigation of its stereotypical behaviors clarified the dynamics and neuronal control mechanisms producing these behaviors [40,41,44,45,46,47].

## 3. Prototype Design and Development

Drawing inspiration from the octopus, we propose a soft biomimetic arm that employs embedded, pneumatically actuated chambers instead of muscles in a configuration analogous to that of the octopus arm muscles to achieve similar motion capabilities.

### 3.1. Design of the Soft Arm Prototypes

Two soft biomimetic arm prototypes were designed and built within the scope of this work: A single-segment arm and a three-segment arm. The former was developed as a preliminary proof-of-concept prototype to study the design, fabrication, and functional characteristics of the proposed concept. Based on these, the three-segment arm was subsequently developed as a demonstrator prototype of a more integrated and capable system. The arm prototypes are fabricated from a soft, compliant, and elastic material, with a series of linear- and helical-shaped embedded pneumatic chambers. Using solenoid valves to control the flow of compressed air in these chambers allows for bending, extension, and twisting motions of the arms through the elongation of the appropriate, in each case, chambers.

In particular, the single-segment arm (length: 50 mm, weight: 250 g) utilizes four linear chambers, arranged symmetrically at 90° intervals and running along the segment, that recreate the functionality of the longitudinal muscles of the octopus arm. Additionally, a set of helical chambers are placed in the outer surface of the segment and function as the oblique muscles of the octopus arm, providing twisting motions in both the clockwise and counterclockwise directions. For design simplicity, we are not implementing the transverse muscles since the elongation of the arm can be achieved by the simultaneous inflation of all four linear chambers. A series of rigid rings are used to restrict the radial expansion of the chambers when pressurized to implement the desired motions.

**Figure 2:**
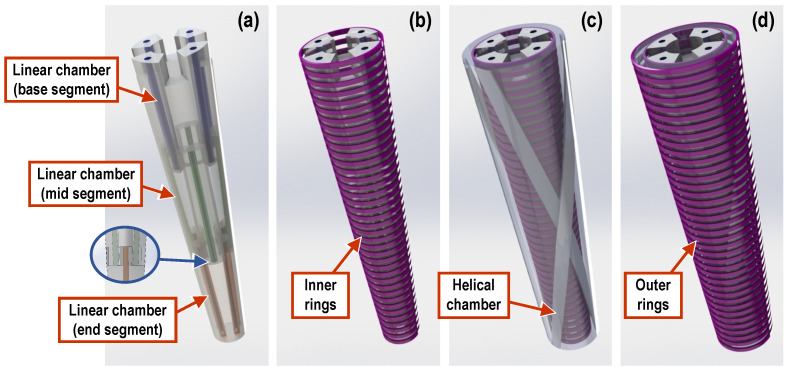
Three-segment soft manipulator arm design elements: (**a**) soft linear chamber assemblies of the three segments (insert shows overlap between consecutive segments), (**b**) set of rigid inner rings constraining the soft linear chambers, (**c**) soft helical chambers, and (**d**) set of rigid outer rings.

**Figure 3:**
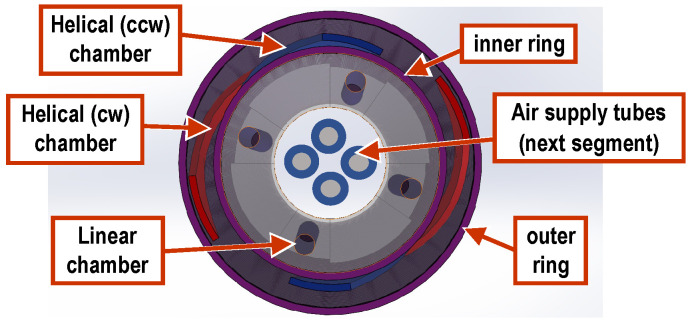
Section view of the second (mid) segment, as seen when looking from the base of the arm towards its tip, showing the arrangement of the different arm design elements.

The three-segment arm, which is designed along similar principles, consists of three segments, each housing four linear chambers as per above, thus providing the ability to bend in three different areas along the length of the arm simultaneously and independently from each other. It is noted that the placement of the linear chambers is rotated by 45° for consecutive segments. In addition, four helical chambers, two clockwise and two counterclockwise, encapsulate the three segments and can be used to twist the arm as a whole. Pressurized air is supplied to each segment via ∅4 mm silicone tubes (∅2 mm inner). In order for the tubes to reach all segments, they must pass through each preceding segment. Hence, eight tubes pass through the first (base) segment and four through the second (mid) one. This requirement, combined with the desire for the arm to have a smooth, continuous outer surface, led to designing the arm to be of a conical shape, with a wide base that gradually tapers at the arm’s tip. As a result, the three individual segments forming the arm are also conical. Because of this conical shape, the four linear chambers inside each segment were positioned to be parallel to the external contour and, hence, tilt towards each other along the tapering of the segment (see Figure 2a). This ensures a homogeneous expansion of the chambers when pressurized. Moreover, in order to eliminate any bending dead zone throughout the arm, each segment is designed to overlap with its neighboring segments by 10 mm (see insert in Figure 2a). Finally, two sets of rigid links, 3d-printed in PLA material, are embedded in the arm, serving a dual function: they are primarily used to constrain the expansion of the chambers to the desired direction and also to minimize the interference between neighboring chambers. More specifically, a set of inner rings is installed on the outer surface of the assembly of the linear chambers (Figure 2b) and separates them from the helical chambers, while a second set of outer rings is installed over the outer edge of the arm (Figure 2d) and provides increased stiffness in the radial direction. In this sense, the rigid rings antagonize the linear chambers, similarly to the way that the transverse muscles antagonize the longitudinal ones in the octopus arm. A section view of the mid-segment, shown in Figure 3, further illustrates the layered arrangement of the elements comprising the arm.

### 3.2. Fabrication of the Soft Arm Prototypes

**Figure 4:**
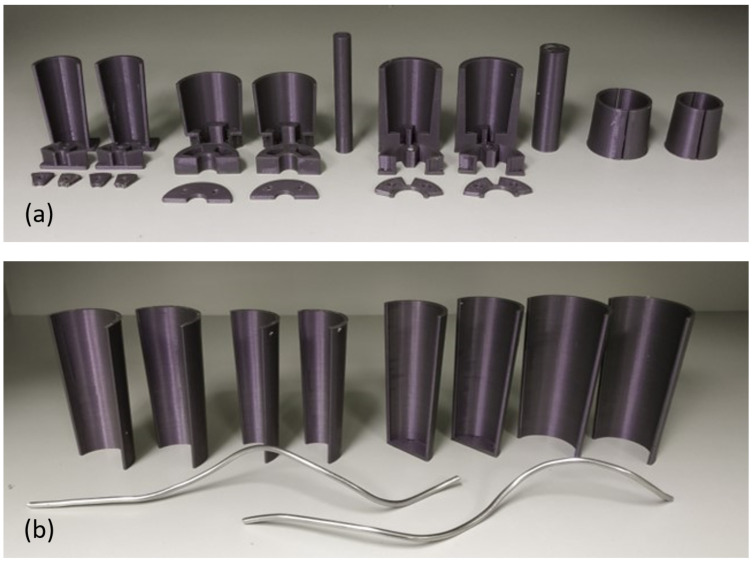
The 3d printed molds employed in the fabrication of the soft manipulator arm: (**a**) linear chamber molds, (**b**) helical chambers molds.

The material used for the fabrication of both prototype arms is a two-component silicone (Ecoflex 00-10 Smooth-On). The molds (see Figure 4), which were used for casting into shape the silicon, were fabricated with PLA material in a 3d printer for each arm design. A room-temperature casting procedure is followed, whereby the liquid silicone mixture is slowly poured from the top of the mold to avoid entrapment of air bubbles that would cause inconsistency in the final part. The pot life of the mixture is 30 min, and it cures over a 4 h period into a 00-10 shore hardness silicone rubber, with a 100% modulus of 8 psi, a tensile strength of 120 psi, and 800% of elongation at break. An alternative option for silicone would be the Dragon Skin 10, with a shore hardness of 10 A, a 100% modulus of 22 psi, a tensile strength of 475 psi, and 1000% of elongation at break. However, the higher shore hardness would require a significantly higher air pressure in order to actuate the arm (from 1 to 2 bar, as operated, to 5 to 6 bar). The selected silicone’s properties, and especially its flexibility, make this silicone one of the most appropriate commercially available options.

To fabricate the three-segment arm, due to the difference in segment dimensions, multiple molds had to be created, one for each segment; hence, multiple castings were necessary in order to bring all segments together in one single arm.

**Figure 5:**
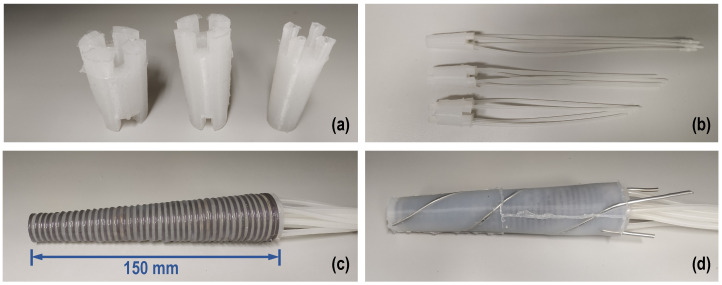
Different stages of the soft multi-segment arm fabrication: (**a**) the three segment cores, (**b**) attachment of the tubes to the linear chambers of the three segments, (**c**) fitting of the inner rings, and (**d**) casting of the helical chambers (the helix-shaped metal inserts are subsequently removed).

**Figure 6:**
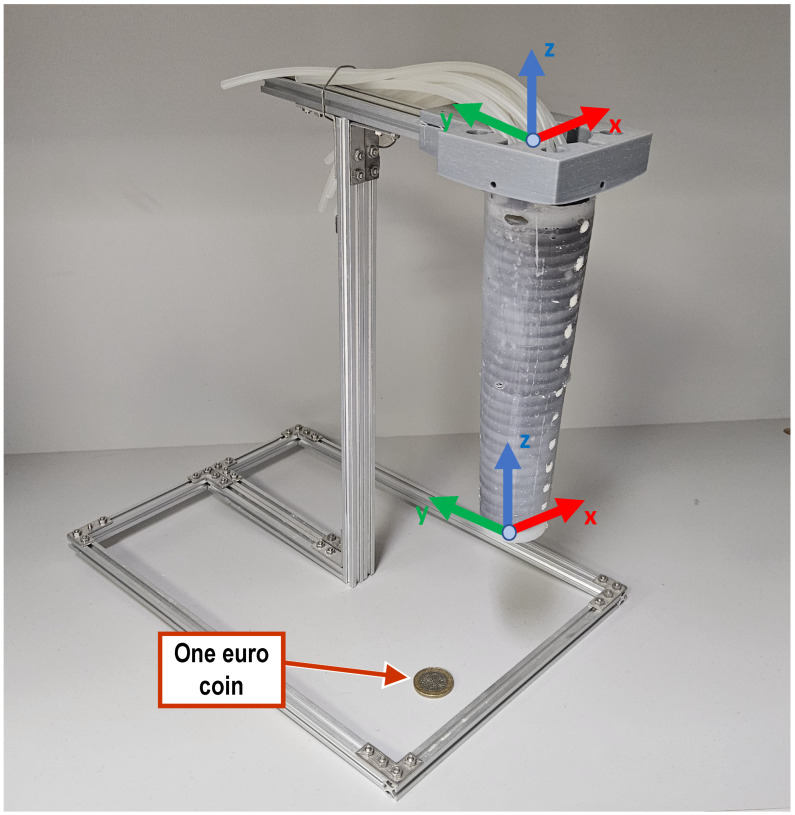
The three-segment soft arm prototype, shown mounted on the stand used during the experiments. The world frame XYZ is located at the base of the arm, while the local xyz frame is attached at the tip of the arm. For size reference, a one-Euro coin (∅23.25 mm) has been included in the photograph.

First, for each of the three segments, the core element (comprising the assembly of the four linear chambers) was cast separately, and silicone tubes were attached to them (see Figure 5a,b) and secured with thin copper wire. All three segments were then joined, each one’s tubes passing through the center of the preceding segments, and the set of inner rings were installed (Figure 5c). The segments were permanently joined together by exploiting the ability of silicone to bond on parts made by the same silicon. Using the molds and the helix-shaped metal inserts shown in Figure 4b, the helical chambers were then cast to surround the three joined segments (Figure 5d). Finally, the outer rings were inserted over the arm and molded into place, creating the complete arm, shown in Figure 6. The overall length of the final prototype is 150 mm, while the diameters of the base and the tip are 49 mm and 35 mm, respectively. The overall mass of the arm is 830 g.

### 3.3. Pneumatic Actuation and Control Unit

**Figure 7:**
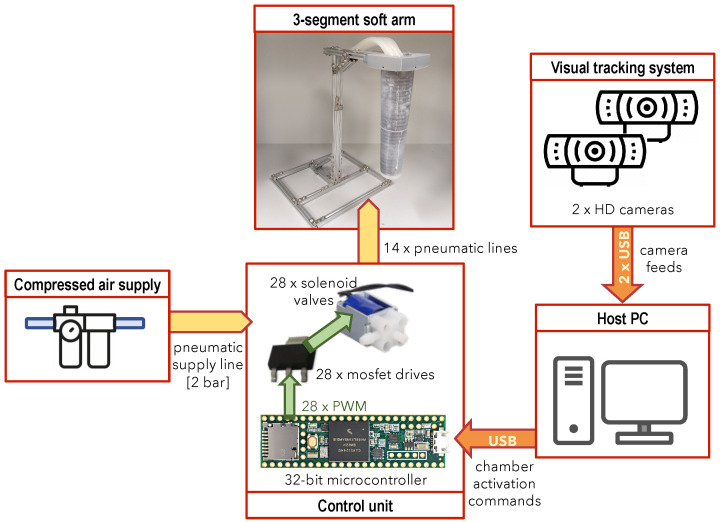
Diagram of the soft arm actuation and control system.

**Figure 8:**
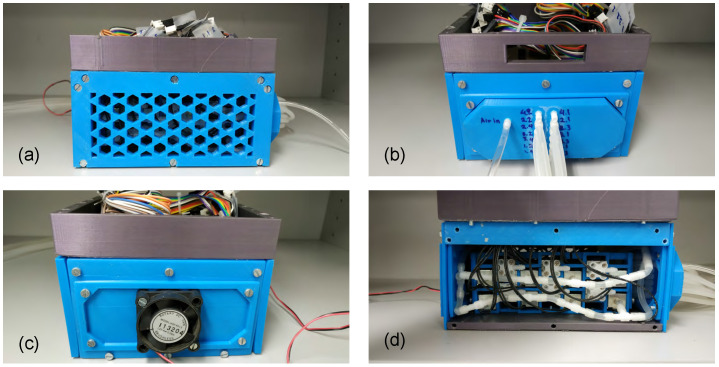
Control unit: (**a**) Side view, (**b**) Front view, (**c**) Back view, and (**d**) Internal view.

A schematic overview of the actuation and control system for the soft arm prototypes is provided in Figure 7, while Figure 8 shows the custom-developed control unit. The latter incorporates a total of 28 miniature solenoid valves (zhv-0519, Zonhen Electric Appliances, Shenzhen, China) that actuate the pneumatic chambers, along with their drive electronics and a 32-bit microcontroller board (Teensy 3.6) that controls their operation. The latter also handles communication via a USB interface, with a host computer that runs the arms control software. These components are housed inside a custom-designed, 3d-printed enclosure that includes fixtures for securing the solenoid valves, a top tray for mounting the main control board, as well as the housing for a small fan providing active cooling to the solenoid valves. The assembled control unit measures 146 mm × 131 mm × 100 mm (length × width × height).

**Figure 9:**
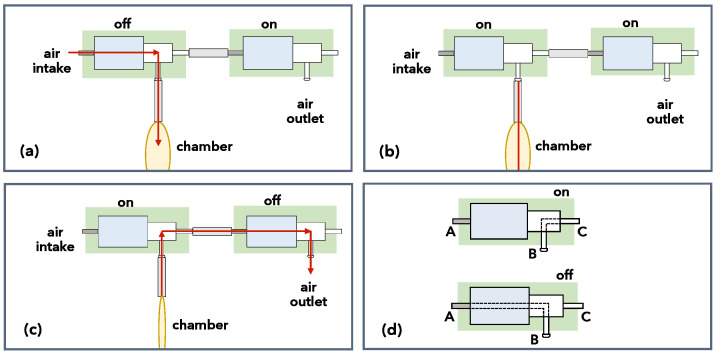
Activation scheme for the pair of solenoid valves controlling air flow to the pneumatic chambers, for (**a**) inflating the chamber, (**b**) maintaining pressurized air in the chamber, and (**c**) deflating the chamber. (**d**) Three-port solenoid valve operation.

Each pneumatic chamber is actuated by a pair of three-port solenoid valves, arranged to achieve, by proper activation, the three necessary operations, namely inserting air into the chamber, retaining the inserted air, and expelling the air from the chamber. This is illustrated in Figure 9, where “on” indicates that a valve is at an energized state that seals the inlet (port A in Figure 9d) and air from the main outlet (port B) can move to the auxiliary outlet (port C), while “off” indicates that air entering the intake is routed to the main outlet. The valve pair is configured so that the inlet port of the first valve is connected to the air supply, while its main outlet is connected to the chamber, and its auxiliary outlet is connected to the inlet port of the second valve. In the case of the helicoidal chambers, the two clockwise chambers are interconnected and thus need only one pair of solenoid valves for their operation, likewise for the counterclockwise helicoidal chambers.

**Figure 10:**
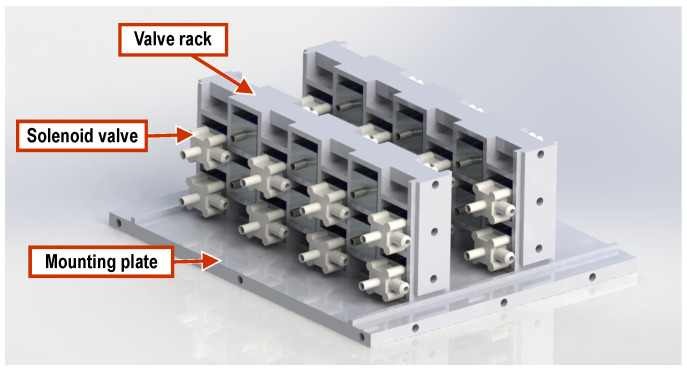
Layout of the solenoid valve assembly, housed inside the control unit.

The valves are mounted on two custom 3d-printed racks, each housing 14 valves inside the control unit (see Figure 10). Compressed air is provided to the control unit by an external source. The housing has vents in its side wall (see Figure 8a) to allow the expelled air from the valves to escape. In addition, a 12 V fan, used to cool the solenoid valves, is mounted on the housing’s rear end (Figure 8c).

**Figure 11:**
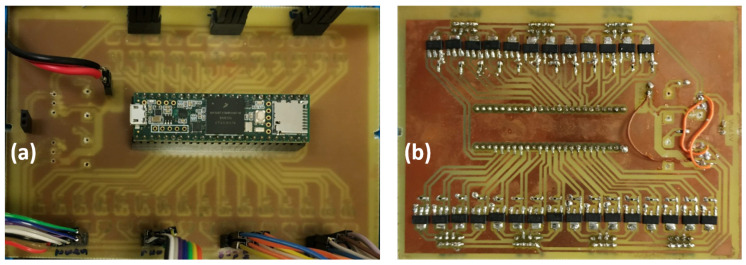
The printed circuit board hosting the arm control electronics: (**a**) top side with the Teensy 3.6 microcontroller board installed, and (**b**) bottom side, with the surface-mounted MOSFET drive circuits (28 in total) of the solenoid valves.

Each solenoid valve is powered with 6 V, and is operated through a dedicated MOSFET (STN4NF03L by STMicroelectronics) circuit that is driven by a 20 Hz PWM signal generated by the microcontroller. By appropriately varying the duty cycle (hence, the duration of the pulse) of the PWM signals driving the valve pair of a particular segment, the rate of inflating/deflating the segment can be varied, thus controlling the speed of arm movements. Due to bandwidth limitations of the solenoid valves’ mechanics, the minimum pulse duration to which the valves can respond is 10 ms (equivalent to a 20% duty cycle of the 20 Hz PWM signal). The control electronics, along with the power, data, and control connectors, are mounted on a custom-made PCB, shown in Figure 11.

### 3.4. Arm Motion Control

**Figure 12:**
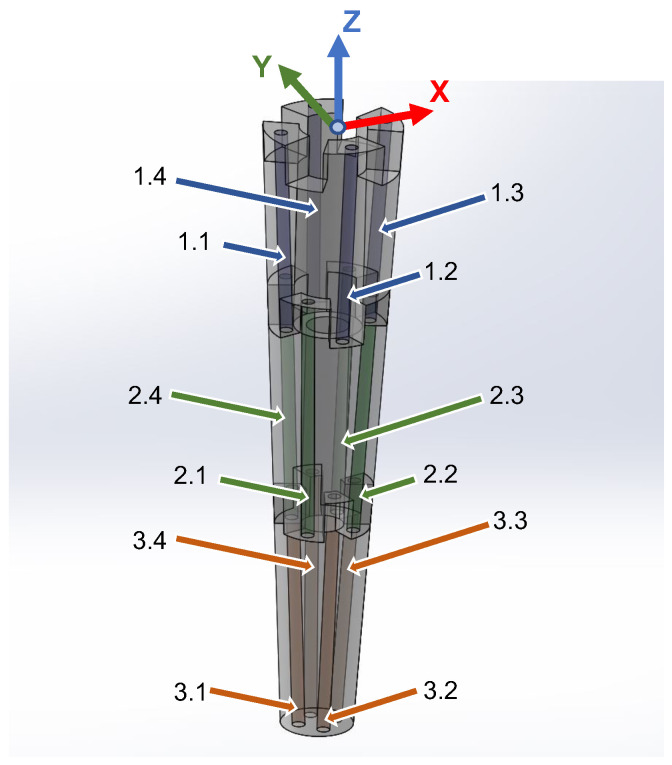
Location and numbering scheme of the linear chambers in the three-segment arm.

The soft arm can move and change its shape through appropriate activation of the linear and helical pneumatic chambers, taking into consideration their topology inside the arm. With regard to the linear chambers, since each segment is rotated by 45° with respect to the previous one, different chambers on each segment must be activated to achieve bending of both segments in the same direction. For example, in order to bend the whole arm in the positive X direction, the chambers identified as 1.1, 2.1, 2.4, and 3.1 in Figure 12 must be inflated. Hence, in this case, for the base and the end segment, only one chamber is inflated, while for the middle segment, two chambers are activated (note that these two chambers must be inflated by an equal amount of air). Similarly, to achieve whole-arm bending in the plane rotated around the *Z*-axis by 45° with respect to the XZ-plane, chambers 1.3, 1.4, 2.3, 3.3, and 3.4 are activated. For arbitrary such rotations of the desired motion plane around the *z*-axis, the chambers that are involved from each segment are inflated to a different degree. Table 1 summarizes the linear chamber activation combinations for attaining the basic shape configurations of the three-segment arm.

For twisting motions, the control is simpler, since the whole arm can be twisted either clockwise or counterclockwise by activation of the respective pair of helical chambers. Twisting can be combined with other motions induced by the activation of linear chambers.

### 3.5. Control Software

Low-level control of the soft arm is implemented through the custom firmware, developed in C language, that is executed on the Teensy 3.6 microcontroller. The firmware is mainly responsible for generating 28 PWM signals driving, through MOSFET gates, the solenoid valves that actuate each one of the pneumatic chambers, according to activation commands received via USB from a host PC at a 100 Hz update rate.

**Figure 13:**
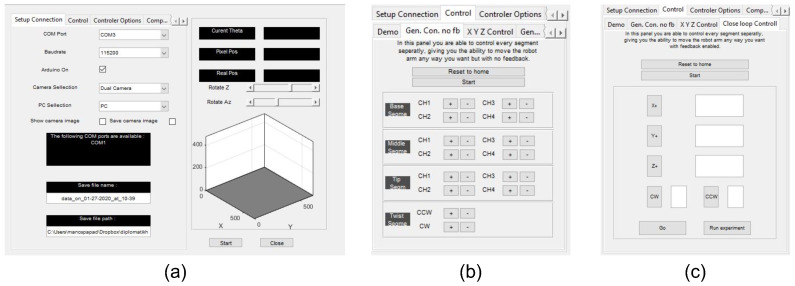
Elements of the Graphical User Interface (GUI) developed for controlling the three-segment arm: (**a**) Startup screen with initialization setup and 3d core pose estimation representation, (**b**) GUI panel for open-loop control of individual chambers, (**c**) GUI panel for closed-loop control.

High-level control of the soft arm is provided through a graphical user interface (GUI) developed in MATLAB and running on the host PC that communicates with the microcontroller. The host PC also receives and processes visual data from the cameras used to track the arm’s motion (see Section 4.2) and to provide feedback for the closed-loop schemes for motion control of the arm (see Section 5.5). The GUI provides a series of panels (see Figure 13) for setting up the communication with the control unit, configuring the cameras, issuing commands to manually set the state of the arm’s chambers, as well as for configuring and executing the closed-loop control schemes. Exporting data, including chamber activation signals, sensor readings, video recordings, etc., to log files for post-processing is also readily supported by the GUI.

## 4. Experimental Setup

Both prototype arms were tested while rigidly mounted on a custom stand constructed using aluminum extrusions (stand footprint: 220 mm × 200 mm stand height: 320 mm), which holds the arm in a vertical position. Figure 6 illustrates the experimental setup along with the global reference frame XYZ (defined to be located on top of the base segment of the arm and oriented so that the *X*- and *Y*-axes are aligned with the stand’s base) and the moving frame xyz (positioned at the tip of the arm so that it has the same orientation as the XYZ frame and is displaced along the *Z*-axis when the arm is at rest).

### 4.1. Force Measurement

**Figure 14:**
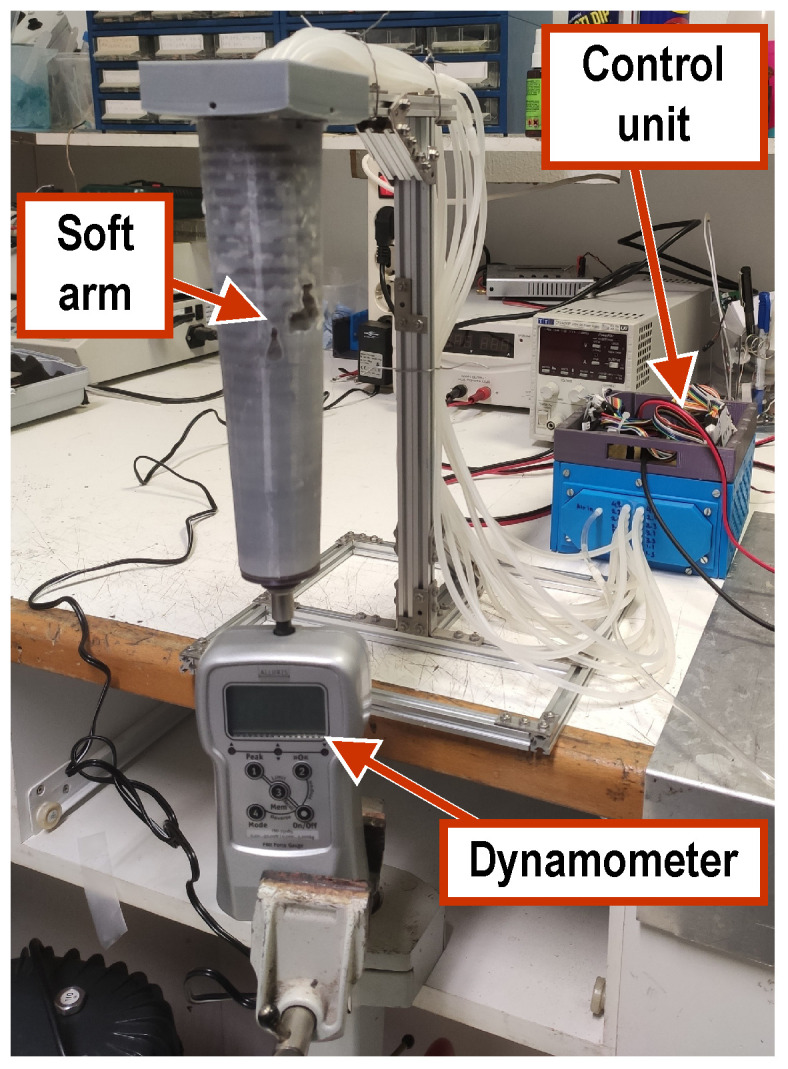
Test setup for force measurement experiments.

A high-precision digital dynamometer (FMI-210A5, Alluris GmbH & Co, Freiburg, Germany) was used to measure, at a 1 kHz rate, the force exerted by the arm, as shown in Figure 14. The dynamometer is placed underneath and in contact with the resting arm. The arm is used in extension mode, where all linear chambers are filled equally with air, and as the chambers expand, the arm presses upon the dynamometer.

### 4.2. Vision-Based Arm Motion Tracking

**Figure 15:**
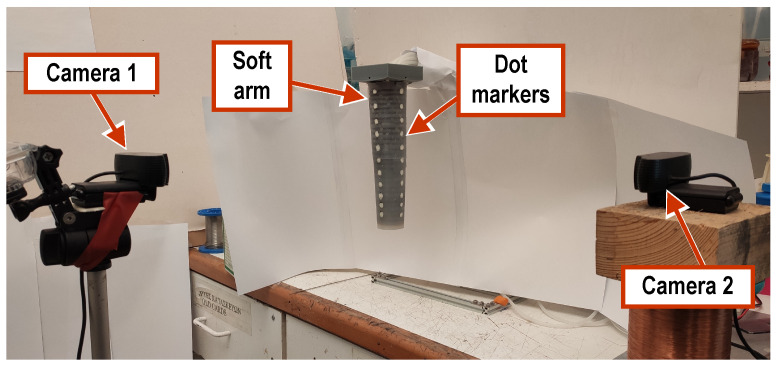
The setup employed for visual tracking of the arm motion.

The position and shape configuration of the arm were tracked using a pair of high-definition cameras (HD Pro C920, Logitech International S.A., Lausanne, Switzerland) placed 30 cm away from the arm and at a 90° angle with respect to each other, as shown in Figure 15. Each camera was calibrated with a checkerboard of known dimensions to determine its extrinsic and intrinsic parameters prior to setting up [48], using the Camera Calibration App of MATLAB [49]. The pair is oriented so that each camera captures the movement of the arm in one plane of movement with regard to the XYZ frame (specifically, Camera 1 for the XZ-plane and Camera 2 for the YZ-plane), using computer vision methods that track two series of painted dot-markers on the arm. Each series consists of 11 such markers positioned at known intervals along a straight line that is parallel to the *Z*-axis when the arm is at its resting state. Combining the images captured by the pair of cameras, a representation of the arm’s configuration in 3d space may be obtained. In particular, calculating the intersections of corresponding markers from the two series yields the 3d coordinates of 11 points forming the center-line of the arm. Real-time processing of the video feeds from the two cameras is performed in MATLAB at a 30 fps rate and is integrated with the GUI described in Section 3.5.

## 5. Experimental Results

Utilizing the experimental setups described in Section 4, tests were conducted to characterize the three-segment arm with respect to the forces it is capable of exerting and the configurations it can achieve through activation of the linear chambers, while the single-segment arm was tested for its longitudinal extension and twisting capabilities.

### 5.1. Arm Force Experiments

The force generated by the three-segment arm in the direction of the *Z*-axis was assessed using the experimental setup of Figure 14. Pressurized air was incrementally supplied to all the arm’s linear chambers by providing a sequence of 5 discrete 10 ms activation pulses, where 7 s were allowed between consecutive pulses. The exerted force was measured 6 s after each pulse to ensure the absence of any transients. This test was repeated for different air pressures provided to the system, ranging from 0.1 to 0.5 bar.

**Figure 16:**
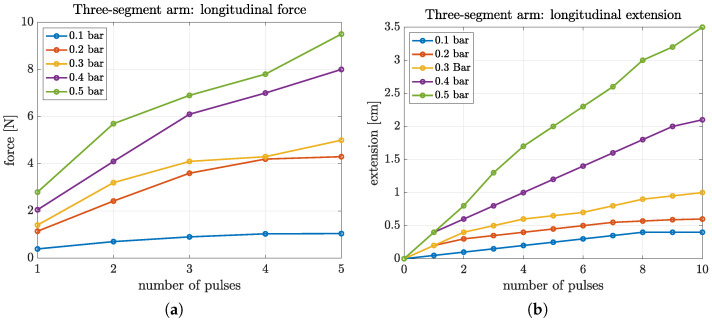
Measurements of (**a**) the exerted force, and (**b**) the extension of the three-segment arm during experiments involving the simultaneous activation of all the arm’s linear chambers.

The average values at steady-state of the thus measured forces are summarized in Figure 16a. It can be seen that the force exerted by the arm increases with the number of air input pulses, as well as with the pressure of the air provided to the system, reaching a maximum of approximately 10 N. Moreover, we observe that the exerted force magnitude is not linearly proportional to the number of input pulses; this is an anticipated result, since the pressure provided by the external system remains constant, while the pressure inside the chambers increases, resulting in a lower pressure difference between them and thus the pressure increase in a chamber for the constant time pulses drops.

### 5.2. Arm Extension Experiments

The vision-based arm tracking setup in Figure 15 was used to measure the elongation characteristics of the two arm prototypes. For the three-segment arm, the arm extension along the *Z*-axis was measured with respect to the pressure of the air intake (ranging from 0.1 to 0.5 bar) and the number of pulses (1 to 10) provided to the linear chambers. The pulsing inputs were applied following an activation sequence like the one used in the force measurement tests. The results are summarized in Figure 16b. It can be seen that increasing the supply pressure affords a significant increase in arm elongation (up to approximately 24% compared to its non-inflated state).

**Figure 17:**
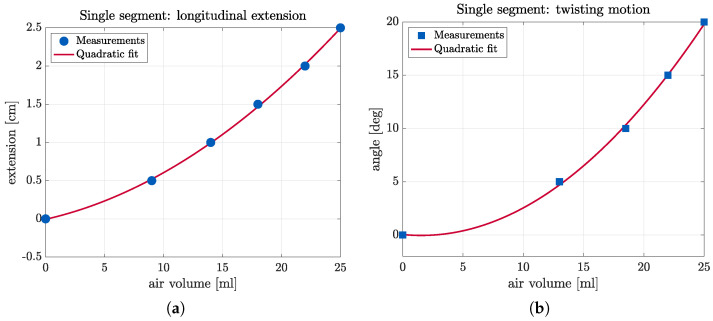
Measurements of (**a**) the longitudinal extension, and (**b**) the twist angle of the single-segment arm prototype as a function of the input air volume.

The longitudinal extension of the single-segment arm was assessed by a different method, namely using a syringe to manually insert known volumes of air in the segment’s four linear segments. Results, shown in Figure 17a, suggest a quadratic relationship between the volume of air inserted in the arm’s chambers and its elongation. It can be seen that the arm is elongated by 2.5 cm (i.e., by 50%) through the injection of a total of 25 mL of air in its four linear chambers. Compared to the experimental results shown in Figure 16b, where, instead of inserting a known volume of air, pressurized air is inserted in the chamber in pulses, we can deduce that the slower increase of displacement based on the number of pulses is due to the lowering pressure difference between the pressure of the air in the chamber and the pressure provided to the control unit, which results in a decrease of the volume of air inserted in each successive pulse.

### 5.3. Arm Twist Experiments

To determine the arm’s twisting capability, tests were conducted with the single-segment prototype mounted on the stand shown in Figure 15. During this experiment, the air was inserted with a syringe in the helical chambers, and measurements were taken of the arm’s twist angle (rotation around the *Z*-axis) as a function of the volume of air inserted.

Inserting air in the helical chambers inflates them, and, due to the restriction of expansion in the lateral direction, the chambers expand by increasing their length. This expansion results in a twisting motion. The results, shown in Figure 17b, indicate a quadratic relation of the twist angle with respect to the volume of air inserted, similar to the arm extension experiments.

### 5.4. Composite Arm Movements

In this set of experiments, the motion tracking setup of Figure 15 was used to capture and analyze the movement of the arm for various open-loop activation patterns of the linear chambers, with the pressure of the air supply set to 1.0 bar.

**Figure 18:**
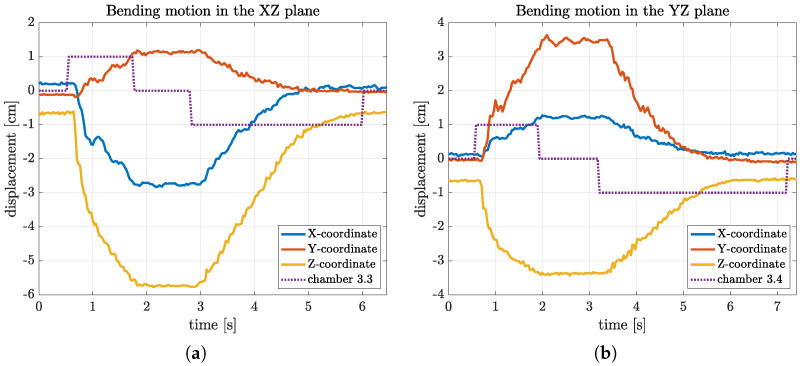
Experimental results for bending motion under activation of a single chamber of the end segment: (**a**) bending motion in the XZ-plane (activation of chamber 3.3), and (**b**) bending motion in the YZ-plane (activation of chamber 3.4).

Results from an indicative such experiment are provided in Figure 18a. Solid lines correspond to the displacement of the arm’s tip along the *X*, *Y*, and *Z* axes, resulting from the sequence of activation, indicated with the dotted line, of a single linear chamber of the end segment, namely the segment identified as 3.3 in Figure 12 (all the other chambers are deflated throughout the experiment). Specifically, from 0.5 s to 1.7 s, the valve pair is activated so that compressed air is inserted into the chamber (Figure 9a) via continuous pulsing. This results in a bending motion of the arm’s lower part, occurring, as anticipated, predominantly on the XZ-plane (there is also some limited displacement along the *Y*-axis). At 1.7 s, the valve pair is activated to seal the chamber (Figure 9b), thus stabilizing the tip. Finally, at 2.8 s, the valve pair is activated to deflate the chamber (Figure 9c), and the arm returns, within about 2.5 s, to its original, non-actuated configuration. An analogous response is recorded in the results shown in Figure 18b, taken from a similar experiment that involved activation of a different chamber of the end segment (namely the chamber identified as 3.4 in Figure 12), resulting in a bending motion on the YZ-plane.

**Figure 19:**
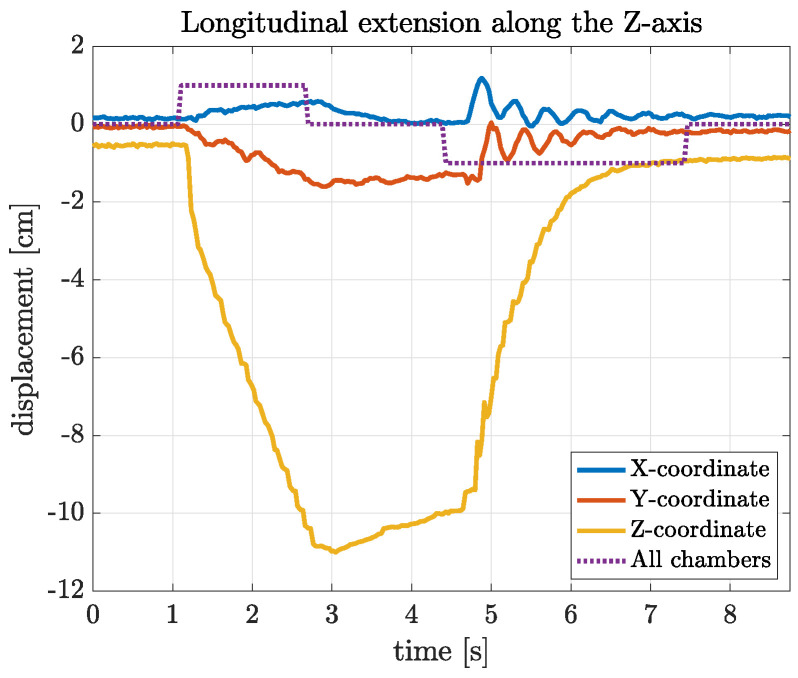
Results for the motion of the arm’s tip during an experiment involving the longitudinal extension of the soft robot, with the pressure of the air supply set to 1.0 bar.

Figure 19 shows the data from an experiment involving the longitudinal extension of the arm through the activation of all 12 of the linear chambers with the same pattern (indicated with the dotted line). It can be seen that the tip of the arm is displaced along the *Z*-axis by as much as 11 cm (i.e., the arm is elongated by approximately 72%) in about 1.8 s. During this extension, there is very limited displacement along the *X*- and *Y*-axes. When the valves are commanded, at 4.5 s, to release the air from the chambers, the arm returns to its resting, non-actuated position within 2.6 s, exhibiting a certain number of oscillations. These can be attributed to the fact that when the chambers are depressurized, the arm is at its lowest stiffness.

**Figure 20:**
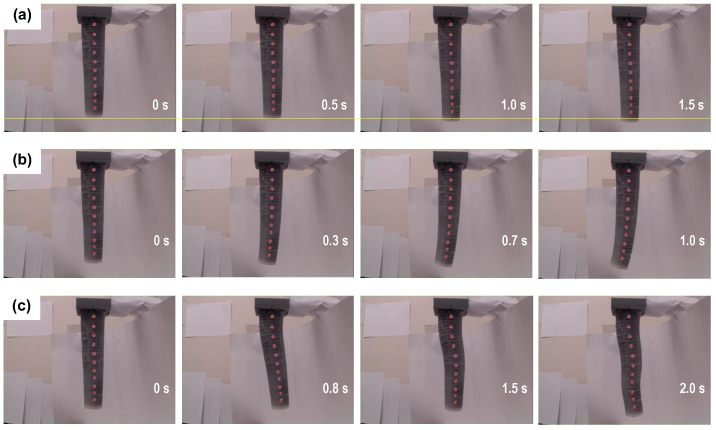
Series of frames from experiments involving (**a**) longitudinal extension, (**b**) whole-arm bending, and (**c**) S-shaped bending, of the three-segment soft arm.

Finally, Figure 20 shows three indicative sequences of frames from various tests with the three-segment arm. The last sequence (Figure 20c) is taken from an experiment demonstrating the capability of the soft arm for attaining S-shaped configurations through the bending of consecutive segments on opposite sides by the activation pattern registered in the last row of Table 1.

### 5.5. Closed-Loop Arm Control

Experiments were also conducted where the external stereo vision system was employed to provide a three-dimensional representation of the arm in real time. The latter can be used as feedback for closed-loop control of, e.g., the position of the arm’s tip, the twisting angle, or the motion plane and extent of bending.

**Figure 21:**
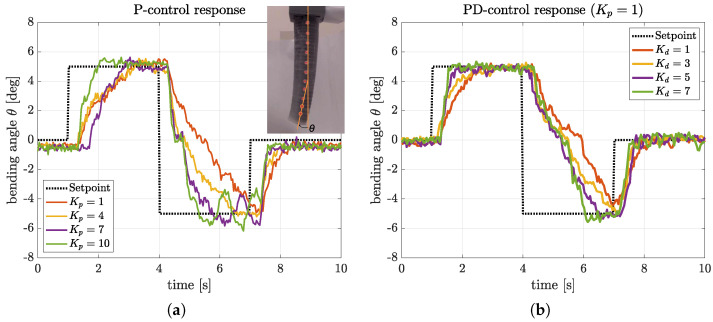
Bending angle response for the three-segment arm under closed-loop control: (**a**) P-controller for different values of the proportional gain $K_{p}$, (**b**) PD-controller for fixed $K_{p}$ and different values of the derivative gain $K_{d}$.

Here, we present results from experiments for closed-loop control of the bending angle θ (see inset in Figure 21a), defined as the angle between the *Z*-axis of the global frame (arm base) and the *z*-axis of the local frame (arm tip), during whole-arm bending motion on the YZ-plane (see Table 1).

Two types of controllers were considered, namely a proportional-only (P-control) and a proportional-derivative (PD-control) one, using as input the error between the setpoint and the vision-estimated value of θ. The output of the controller is used to specify the duty cycle of the PWM signals activating the solenoid valve pairs of the appropriate chambers, namely chambers {1.2, 2.1, 2.2, 3.2} for θ>0 and chambers {1.4, 2.3, 2.4, 3.4} for θ<0.

Initial experiments focused on the effect of the P-controller gain $K_{p}$ on the obtained response, involving both positive and negative values for the bending angle setpoint. The results, summarized in Figure 21a, indicate that the P-controller can regulate the bending angle to the specified setpoint with good accuracy. As anticipated, increasing the control gain results in a faster overall response. However, for large $K_{p}$ values ($K_{p}=7$ and $K_{p}=10$ in Figure 21a) there are undesirable oscillations, as well as a small dead zone near the zero degrees mark when the system moves from a positive to a negative setpoint.

As shown in Figure 21b, the PD-controller yields marked improvements, where the introduction of the error-derivative control action, with appropriate gain settings (e.g., for $K_{p}=1$, $K_{d}=5$), allows for efficient damping of the oscillations while maintaining a fast and accurate response. Given, in particular, the minimal steady-state error thus attained, we opted against including an integral-error term to the controller to avoid degradation of the transient response.

## 6. Discussion and Conclusions

We have presented the development of an octopus-inspired soft arm prototype that closely emulates the musculature of an octopus, utilizing pneumatically actuated chambers to achieve comparable motion capabilities. Linear chambers mimic the longitudinal muscles, enabling bending and elongation, while helical chambers replicate the oblique muscles, allowing for twisting motions—a novel feature rare among similar prototypes. This bi-directional twisting ability enhances the arm’s versatility and is achieved through both clockwise and counterclockwise helical chambers. Rigid 3d-printed rings function similarly to the transverse muscles by constraining radial expansion, ensuring precise, directed movement. The three-segment arm incorporates a conical, overlapping design to avoid bending dead zones, with appropriately rotated and tapered chambers for uniform actuation. Compressed air is delivered through internal silicone tubes, making the design robust and capable of complex, multi-segment motion akin to the natural dexterity of an octopus arm, as demonstrated through experimental validation studies. Moreover, we have demonstrated the efficacy of simple, model-free closed-loop controllers to position the arm tip with speed and accuracy.

A noteworthy finding to emerge from our studies is that thanks to the durability of the silicone employed, a carefully fabricated mechanism without air leaks allows the arm to hold any given shape indefinitely, provided external forces do not change. Even while resisting varying external forces, this enables the arm to perform consistently in any orientation and maintain its functionality.

It is noted that the conventional casting methods used to fabricate our prototypes are quite involved and are prone to inconsistencies, primarily due to the multi-step process required to accommodate the arm’s complex morphology. To address this issue, one promising alternative is the use of multi-material 3d printing technologies, which could enable the creation of the entire arm in a single, streamlined stage. This would not only eliminate inconsistencies in the arm’s soft materials but also allow for the seamless integration of pneumatic circuits and other functional components directly into the structure. Such advancements could significantly enhance the arm’s precision, durability, and overall controllability while also reducing production time and effort.

While the foundation for the creation of a functional pneumatic arm has been established in this study, there are still considerable challenges that need to be addressed, opening up new and interesting research directions. One priority is the integration of embedded sensors to measure the arm’s movements directly. For instance, flex sensors could be incorporated into the inner and outer surfaces of the arm, enabling precise deformation measurements at known locations. Such data would allow the development of real-time pose estimation models, eliminating the need for external tracking systems and greatly improving the control, precision, and application scope of the arm.

Exploring alternative chamber shapes and placements is another promising avenue to expand the variety of achievable motion patterns. Adjustments to the configuration of linear and helical chambers could enhance dexterity and efficiency. Additionally, experimenting with different actuation fluids, such as water or oil, could reveal how these fluids affect the arm’s performance, particularly in terms of force output, responsiveness, and environmental suitability.

Miniaturization of the arm holds substantial potential, particularly for medical applications. A smaller arm would reduce resource demands while maintaining functionality. Scaling down components, including solenoid valves and air compressors, could enable the creation of a soft robotic arm small enough for laparoscopic procedures. Its compliance and soft materials would provide a safer alternative for interacting with delicate tissues, reducing the risk of damage during surgical operations.

Conversely, a scaled-up version of the arm could be explored for applications requiring safe, dexterous manipulation of larger objects, e.g., in manufacturing, where it could handle delicate assembly tasks; in agriculture, for gentle crop harvesting; or in challenging environments like disaster zones, where its flexibility could aid in search-and-rescue missions. Moreover, the arm’s compliance and adaptability could make it valuable for space exploration, where soft robotic systems are particularly advantageous for interacting with fragile equipment or unpredictable environments.

## Figures and Tables

**Table 1:** Examples of linear chamber activation combinations for attaining different shape configurations of the three-segment arm. Filled circles denote the inflated chambers. Chambers are identified according to Figure 12.

	Linear Chamber											
Direction	3.1	3.2	3.3	3.4	2.1	2.2	2.3	2.4	1.1	1.2	1.3	1.4
whole-arm bending												
+X	●	○	○	○	●	○	○	●	●	○	○	○
−X	○	○	●	○	○	●	●	○	○	○	●	○
+Y	○	●	○	○	●	●	○	○	○	●	○	○
−Y	○	○	○	●	○	○	●	●	○	○	○	●
whole-arm longitudinal extension												
−Z	●	●	●	●	●	●	●	●	●	●	●	●
composite configurations												
S-shape	●	○	○	○	○	●	●	○	●	○	○	○
Ƨ-shape	○	○	●	○	●	○	○	●	○	○	●	○

## Data Availability

Data are contained within the article.
